# The Dopamine D2 Receptor Gene in Lamprey, Its Expression in the Striatum and Cellular Effects of D2 Receptor Activation

**DOI:** 10.1371/journal.pone.0035642

**Published:** 2012-04-26

**Authors:** Brita Robertson, Icnelia Huerta-Ocampo, Jesper Ericsson, Marcus Stephenson-Jones, Juan Pérez-Fernández, J. Paul Bolam, Rochellys Diaz-Heijtz, Sten Grillner

**Affiliations:** 1 Department of Neuroscience, Karolinska Institutet, Stockholm, Sweden; 2 MRC Anatomical Neuropharmacology Unit, Department of Pharmacology, University of Oxford, Oxford, United Kingdom; 3 Neurolam Group, Department of Functional Biology and Health Sciences, Faculty of Biology, University of Vigo, Vigo, Spain; University of Waterloo, Canada

## Abstract

All basal ganglia subnuclei have recently been identified in lampreys, the phylogenetically oldest group of vertebrates. Furthermore, the interconnectivity of these nuclei is similar to mammals and tyrosine hydroxylase-positive (dopaminergic) fibers have been detected within the input layer, the striatum. Striatal processing is critically dependent on the interplay with the dopamine system, and we explore here whether D2 receptors are expressed in the lamprey striatum and their potential role. We have identified a cDNA encoding the dopamine D2 receptor from the lamprey brain and the deduced protein sequence showed close phylogenetic relationship with other vertebrate D2 receptors, and an almost 100% identity within the transmembrane domains containing the amino acids essential for dopamine binding. There was a strong and distinct expression of D2 receptor mRNA in a subpopulation of striatal neurons, and in the same region tyrosine hydroxylase-immunoreactive synaptic terminals were identified at the ultrastructural level. The synaptic incidence of tyrosine hydroxylase-immunoreactive boutons was highest in a region ventrolateral to the compact layer of striatal neurons, a region where most striatal dendrites arborise. Application of a D2 receptor agonist modulates striatal neurons by causing a reduced spike discharge and a diminished post-inhibitory rebound. We conclude that the D2 receptor gene had already evolved in the earliest group of vertebrates, cyclostomes, when they diverged from the main vertebrate line of evolution (560 mya), and that it is expressed in striatum where it exerts similar cellular effects to that in other vertebrates. These results together with our previous published data (Stephenson-Jones *et al.* 2011, 2012) further emphasize the high degree of conservation of the basal ganglia, also with regard to the indirect loop, and its role as a basic mechanism for action selection in all vertebrates.

## Introduction

For an animal to achieve its goal it has to select between competing actions. A group of subcortical nuclei, the basal ganglia, play a key role in this process in vertebrates [1]–[5]. The neuronal architecture underlying selection is evolutionarily highly conserved, as all components of the basal ganglia, including the striatum and homologues of the globus pallidus, substantia nigra (*pars compacta* and *reticulata* (SNr)) and the subthalamic nucleus (STN), have been identified in the lampreys [6] [7], the phylogenetically oldest vertebrates that diverged from the main vertebrate line 560 million years ago [8]. Furthermore, the lamprey striatum receives input from the cortex/pallium and thalamus [9] and GABAergic striatal neurons in both lampreys [10] and mammals [11] display inward rectification, a key feature critical in the expression of basal ganglia function.

Information in the striatum is transmitted monosynaptically by GABAergic substance P-expressing projection neurons to the output nuclei of the basal ganglia, the globus pallidus *interna* (GPi) and the SNr (direct pathway) or polysynaptically, by enkephalin-expressing neurons projecting to the GP *externa* (GPe) and the STN and thence to the output nuclei (indirect pathway; see [7], [12]). A difference between mammals on one side and lampreys and birds on the other, however, is that GPi and GPe are intermingled in the latter group but clearly distinct in mammals [7], [13].

The main modulatory input to the striatum is dopaminergic in both lamprey [9] and mammals (see [14]). The functional role of dopamine in lampreys was recently studied through dopamine depletion with methyl-phenyl tetrahydropyridine (MPTP). This caused a marked hypokinesia, as in Parkinson's disease, an effect that was counteracted by treatment with dopamine agonists [15]. Dopamine receptors constitute two structurally and functionally different classes of dopamine receptors; the D1 receptor class (D1A/D1 and D1B/D5) and the D2 class (D2, D3 and D4) (see [16]). The D1 and D2 classes are thought to have different evolutionary origin and are considered to have independently and convergently acquired the ability to bind dopamine [17]. In mammals, dopaminergic modulation of direct pathway neurons is dependent on dopamine D1 receptors, whereas modulation of indirect pathway neurons is mediated by D2 receptors. In lamprey, only a partial D1-like receptor gene has been cloned [18] and shown to be expressed in a subpopulation of striatal neurons [19]. Whether a D2 receptor is expressed in cyclostomes and if so, whether this receptor is functional is the subject of the present study. D2 receptors have previously only been identified from the level of teleosts to primates (e.g. [17], [20]–[23]). The presence of a functional D2 receptor would provide important additional evidence of the evolutionary conservation of the receptor subtype and the operation of the basal ganglia circuitry. Here, we identify a lamprey D2 receptor gene and show its expression in a subpopulation of neurons in the striatum, where tyrosine hydroxylase-positive (TH) boutons make synaptic contact with striatal dendrites. In addition, we show that a D2 receptor agonist reduces action potential discharge of striatal neurons, in a similar manner to that observed in other vertebrates.

## Results

### Cloning and sequence analysis of lamprey dopamine D2 receptor

In order to identify a lamprey dopamine D2 receptor, the human dopamine D2 receptor long chain (accession number NM_000795) was aligned using BLAST against the sea lamprey (*Petromyzon marinus*) genome (http://blast.ncbi.nlm.nih.gov). A lamprey D2 receptor-like sequence sharing an 80% identity with the human sequence was assembled and used as template. Table 1 shows the different primers used to obtain the 5′ and 3′ sequences. In addition, we used the 5′- and 3′-RACE PCR to amplify the respective sequences including the unconserved amino terminus and to verify the start and stop codons. At least two sets of primers were used to amplify the different regions.

**Table 1:** Primers (5′ to 3′) used for the cloning of the lamprey dopamine D2 receptor gene.

	Forward primer	Reverse primer
**Fw-115/Re170**	ATGCTTGCGGAGCTGCAGAGA	TCGCCGTCTCGCGTGAAAAG
**Fw-80/Re170**	GCACATTTTGCCCAACCGCGA	TCGCCGTCTCGCGTGAAAAGT
**Fw128/Re767**	TGCTCATATGCCTCATCGTC	TCAAGCTTTGCACAATCGTC
**Fw161/Re420**	TCGTCTGCATCGCCGTCT	GCTCTACAACACCCGCTACCG
**Fw407/Re643**	TCGCGATGCCCGTGCTCTACAAC	ATCTACGTGGTCCCTGCGCAAGCG
**Fw431/Re668**	CCCGCTACCGGTCTCGACGGAGAGT	GCAAAAGGGTCAACGCCAAGCGC
**5′ RACE Re410**		CGATGCCCGTGCTCTACAACACCC
**Fw469/Re957**	TCTGTGGTTTGGGTCCTGTC	CCAACAACACTCGCTCGG
**Fw431/Re1497**	CCCGCTACCGGTCTCGACGGAGAGT	GTTCCGCAAGGCCTTCATTAAAA
**Fw951/Re1492**	GGCGAGCCAACAACACTC	ATAGAGTTCCGCAAGGCCTT
**Fw1289/Re1497**	CGCAACAGAGAGAGAAGAAGGCGACTC	GTTCCGCAAGGCCTTCATTAAAA
**Fw1277/Re1492**	ATCGGAATTTTTCGCAACAG	ATAGAGTTCCGCAAGGCCTT
**3′ RACE Fw1290**	GCAACAGAGAGAGAAGAAGGCGACTCA	
**3′ RACE Fw1294**	CAGAGAGAGAAGAAGGCGACTCAGATGC	

Fw, forward; Re, reverse.

The cloned D2 receptor cDNA sequence of the lamprey comprises an open reading frame of 1533 nucleotides encoding a protein of 511 amino acids. (Figure S1). The putative start codon was identified by being embedded within a strong consensus motif for a ribosomal binding site; with a G following ATG (position +4) and a purine, G, three nucleotides upstream (position −3) (see Figure S1; [24]). In addition, the BLAST analysis showed that the length of the lamprey D2 receptor amino terminus is similar to that of other vertebrates.

**Figure 1:**
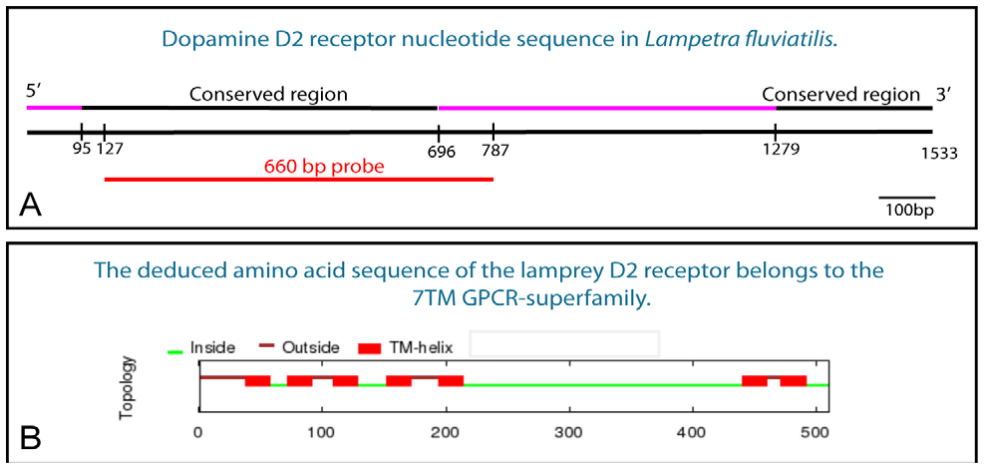
The lamprey dopamine D2 receptor. **A.** A schematic drawing of the lamprey dopamine D2 receptor nucleotide sequence showing the conserved regions (black) at the 5′ and 3′ ends, respectively, and the site from which the riboprobe (660 bp, red) was made. **B.** The seven transmembrane domains (TM-helix) are all located at the conserved parts of the dopamine D2 receptor. The deduced amino acid sequence of the receptor shows that it belongs to the GPCR-superfamily.

**Figure 2:**
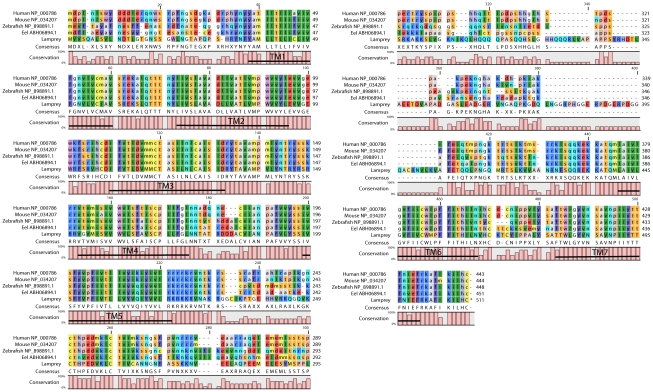
Alignment of dopamine D2 receptor amino acid sequences. Alignment of the deduced amino acid sequence from the lamprey dopamine D2 receptor with those from *Homo sapiens*, *Mus musculus*, *Danio rerio* and *Anguilla anguilla*. The seven transmembrane domains are indicated.

The D2 receptor sequence contains two highly conserved regions. One conserved region was found in the 5′ region comprising 601 nucleotides and a second at the 3′ end comprising 254 nucleotides (Figure 1A). In between the two conserved regions, a sequence of 586 nucleotides, comprising the third intracellular loop, shows little identity with other vertebrate D2 receptor sequences (Figures 1A, B, see also Figure 2). An additional unconserved region was found at the 5′ end comprising 95 nucleotides.

#### Transmembrane domains

The deduced amino acid sequence exhibited the typical profile of a seven transmembrane (TM) G-protein coupled receptor (GPCR). The first five TM domains were located at the conserved 5′ end, and TM 6 and 7 were located at the conserved 3′ end (Figures 1B and 2). As apparent in Figure 2, the lamprey dopamine D2 receptor shares a very high identity within all TM domains, with the second and third domains showing an 100% identity, when compared with the TM domains of *Homo sapiens*, *Mus musculus*, *Danio rerio* and *Anguilla anguilla*. Interestingly, when an amino acid differs in the lamprey it mostly also differs in one or several of the other species examined. The entire amino acid sequence in the lamprey shows around 60% identity with other vertebrate D2 receptor protein sequences.

The lamprey protein contains highly conserved amino acid residues that are thought to line the dopamine binding pocket within the hydrophobic TM domains, in particular valine residues in TM2, an aspartic acid residue in TM3, two serine residues in TM5, and a highly conserved WLPFF sequence and a histidine residue in TM6 [25], [26]. Many characteristic features of GPCRs were identified in the protein sequence of the lamprey D2 receptor, such as a conserved DRY sequence at the end of TM3, which is mandatory for the interaction of the receptor with G proteins [27]. The lamprey protein also contains several consensus phosphorylation sites for protein kinases (PKC, PKA and CKII) and a consensus N-glycosylation site in the NH_2_-terminus (see Figure S1; [27]. In addition, it showed the typical features of the D2 class of dopamine receptors such as a relatively long third intracellular loop and the carboxyl-terminal ending with a cysteine residue (Figure 2; [27]–[29]).

#### Phylogenetic analysis

**Figure 3:**
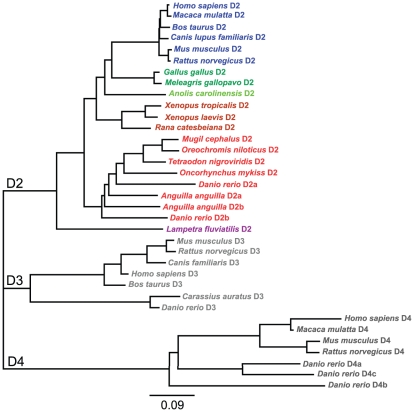
Phylogenetic analysis of vertebrate dopamine receptors of the D2-family. The distant tree was built with the neighbor-joining algorithm from alignments of the D2, D3 and D4 receptor subtypes from several vertebrate species. Data were re-sampled by 1000 bootstrap replicates to determine confidence indices within the phylogenetic tree. The scale bar refers to a phylogenetic distance of 0.09 amino acid substitution per site. The different vertebrate classes are indicated by different colors (mammals, blue; birds, dark green; reptiles, light green; amphibians, brown; fish, red; cyclostomes, mauve). GenBank accession numbers of the sequences are: *Homo sapiens* D2, AAA52761; *Macaca mulatta* D2, XP_001085571; *Bos taurus* D2, DAA22356; *Canis lupus familiaris* D2, AAG34494; *Mus musculus* D2, NP_034207; *Rattus norvegicus* D2, NP_036679; *Gallus gallus* D2, NP_001106761; *Meleagris gallopavo* D2, AAD03818; *Anoils carolinensis* D2, XP_ 003217484; *Xenopus tropicalis* D2, XP_002937871; *Xenopus laevis* D2, CAA51412; *Rana catesbeiana* D2, BAI70438; *Mugil cephalus* D2, AAU87970; *Oreochromis niloticus* D2, AAU87971; *Tetraodon nigroviridis* D2, CAF97490 ; *Oncorhynchus mykiss* D2, CAC87873; *Danio rerio* D2a, AAN87174; *Danio rerio* D2b, AAP94011; *Anguilla anguilla* D2A, ABH06893; *Anguilla anguilla* D2B, ABH06894; *Lampetra fluviatilis* D2, ADO23655; *Mus musculus* D3, 2105315A; *Rattus norvegicus* D3, 1614344A; *Canis lupus familiaris* D3, XP_545106; *Homo sapiens* D3, 1705199A ; *Bos taurus* D3, NP_001179824; *Carassius auratus* D3, ABN70936; *Danio rerio* D3, AAN87173; *Homo sapiens* D4, 1709359A; *Macaca mulatta* D4, XP_001087197; *Mus musculus* D4, 2109259A; *Rattus norvegicus* D4, AAA18588; *Danio rerio* D4a, AAW80614; *Danio rerio* D4b, AAW80615; *Danio rerio* D4c, AAW80616.

We focused our phylogenetic analysis on the D2 receptor family as the BLAST analysis of the lamprey cloned sequence showed identity with the D2 receptor family, but not with the D1 receptor family. The deduced amino acid sequence of the lamprey D2 receptor was aligned with homologous D2 receptor sequences from other known vertebrate representatives. The lamprey sequence was clustered together with other vertebrate D2 sequences, not with the D3 or D4 receptor subtypes, which shows that the lamprey sequence can be assigned to the D2 receptor subtype, based on the primary amino acid sequence (Figure 3). The tree topology further showed that the lamprey sequence is the most distant from the mammalian clade (Figure 3). The cloned lamprey D2 receptor nucleotide sequence is available in the GenBank (accession number HQ331119).

### Dopamine D2 receptor mRNA expression

**Figure 4:**
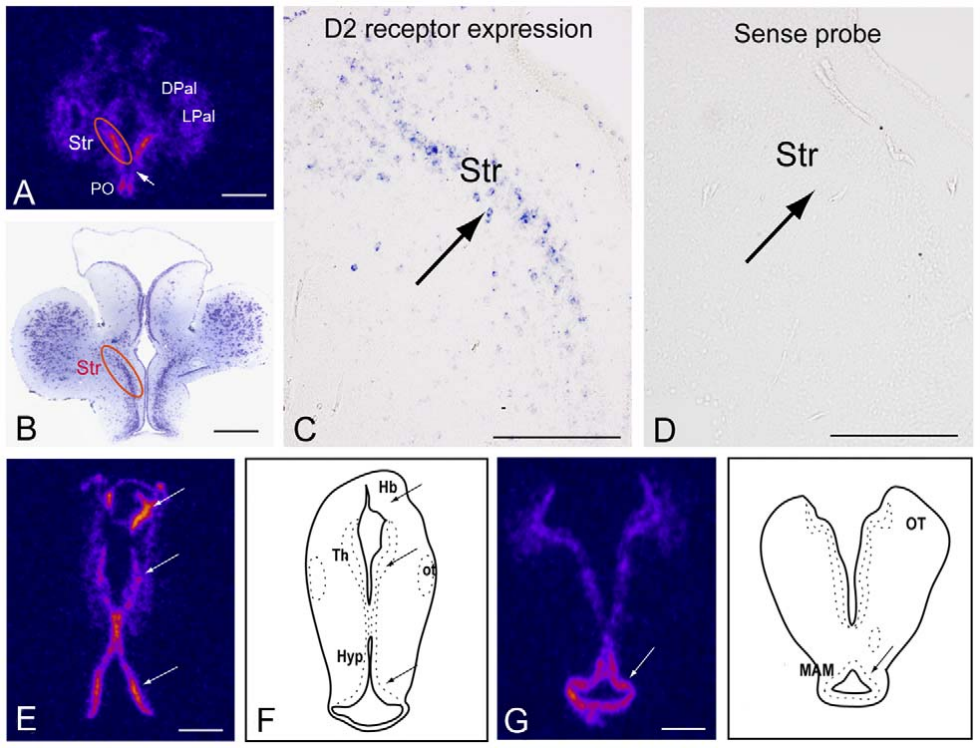
Dopamine D2 receptor expression in the lamprey brain. **A.** **^35^**S-UTP-labeled D2 receptor riboprobe shows strong expression in the striatum. D2 receptor expression is also present in the dorsal and lateral pallium although less strong. Note the absence of receptor expression in the magnocellular preoptic nucleus (arrow). **B.** Nissl stained section from the striatum showing the site of receptor expression in A and C. **C.** DIG-labeled D2 receptor riboprobe expressed in a subpopulation of striatal neurons. **D.** DIG-labeled sense riboprobe showing lack of mRNA expression in the section adjacent to the one in Figure 5C. **E.** **^35^**S-UTP-labeled D2 receptor riboprobe expression in the habenula, thalamus and hypothalamus. **F.** Schematic drawing of the lamprey brain indicating the habenula, thalamus and hypothalamus. **G.** **^35^**S-UTP-labeled D2 receptor riboprobe shows strong expression in the mammillary area. **H.** Schematic drawing of the lamprey brain indicating the mammillary area. The pseudocoloring in **A, E** and **G** indicates signal intensity from low (black/blue) to high (pink/yellow). Abbreviation: *DPal, dorsal pallium; Hb, habenula; Hyp, hypothalamus; LPal, lateral pallium; MAM, mammillary region; ot, optic tract; OT, optic tectum; PO, preoptic nucleus; Str, Striatum; Th, thalamus*. Scale bars, A, B, E and G 500 µm; C and D 200 µm.

The riboprobe used contained the 5′ conserved region, which includes the first five transmembrane domains (see Figure 1A and 2). When the deduced protein sequence of the riboprobe was aligned against other vertebrates using BLAST it showed a 92% identity with the *Anguilla anguilla*, 90% with *Danio rerio*, 86% with *Mus musculus*, and 86% with *Homo sapiens* D2 receptor protein sequences. The specificity of the signals obtained by *in situ* hybridization was confirmed using sense probes (see Figure 4D). Background levels from all brain regions were very low in all sections hybridized with ^35^S-UTP-labeled riboprobes.

In the striatum (Figure 4B), a high level of D2 receptor expression was observed in the whole rostrocaudal extent visualized with either DIG- or *^35^S*-UTP-labeled riboprobes (Figure 4A and C). Only a subpopulation of neurons within the compact striatal cell layer expressed the receptor mRNA (Figure 4C).

The preoptic area also expressed a high level of the D2 receptor (Figure 4A). A small region just ventral to striatum and dorsal to the preoptic area, the magnocellular preoptic nucleus, appears, however, devoid of D2 receptor expression (Figure 4A). Furthermore, neurons in the dorsal and lateral pallium expressed the receptor but at a lower level (Figure 4A). More caudally, high levels of expression are observed in the habenula, thalamus and hypothalamus as well as in the mammillary region (Figure 4E–H).

**Figure 5:**
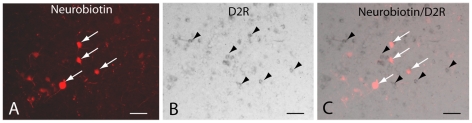
Striatal projection neurons targeting substantia nigra *pars reticulata* (SNr) do not express the D2 receptor. **A.** Striatal neurons retrogradely labeled after injections of neurobiotin into the SNr (white arrows). **B.** DIG-labeled D2 receptor riboprobe expressed in a subpopulation of striatal cells (black arrow heads show examples of positive cells). **C.** Merged image showing the absence of overlap between retrogradely labeled cells and D2 receptor mRNA expression. Scale bars, 100 µm.

Retrograde tracing from the lamprey homologue of the SNr leads to the labeling of only striatal projection neurons expressing substance P and thus belonging to the direct pathway [6]. Consistent with these previous observations we showed retrograde labeling of neurons in the striatum following the injection of neurobiotin in the SNr (Figure 5A). In sections that were double-labeled to reveal the retrogradely labeled neurons and the *in situ* hybridization signal for the D2 receptor, the labels never colocalized (Figure 5A–C; 38 retrogradely labeled neurons analyzed). This finding supports the notion that D2 receptor expressing neurons do not project directly to the output nuclei of the lamprey and are thus likely to be those striatal neurons that project to the GPe.

### Ultrastructural localization of tyrosine hydroxylase-immunoreactive structures

Previous studies have investigated the distribution of dopamine neurons and fibers in the lamprey brain by using immunohistochemical techniques at the light microscope level [9], [30]. In this study we characterized the presence of TH-immunoreactive fibers in the striatum and perfomed a quantitative analysis of dopaminergic profiles at the electron microscopic level to determine synaptic incidence and the nature of the postsynaptic structures in three regions of the lamprey striatum.

**Figure 6:**
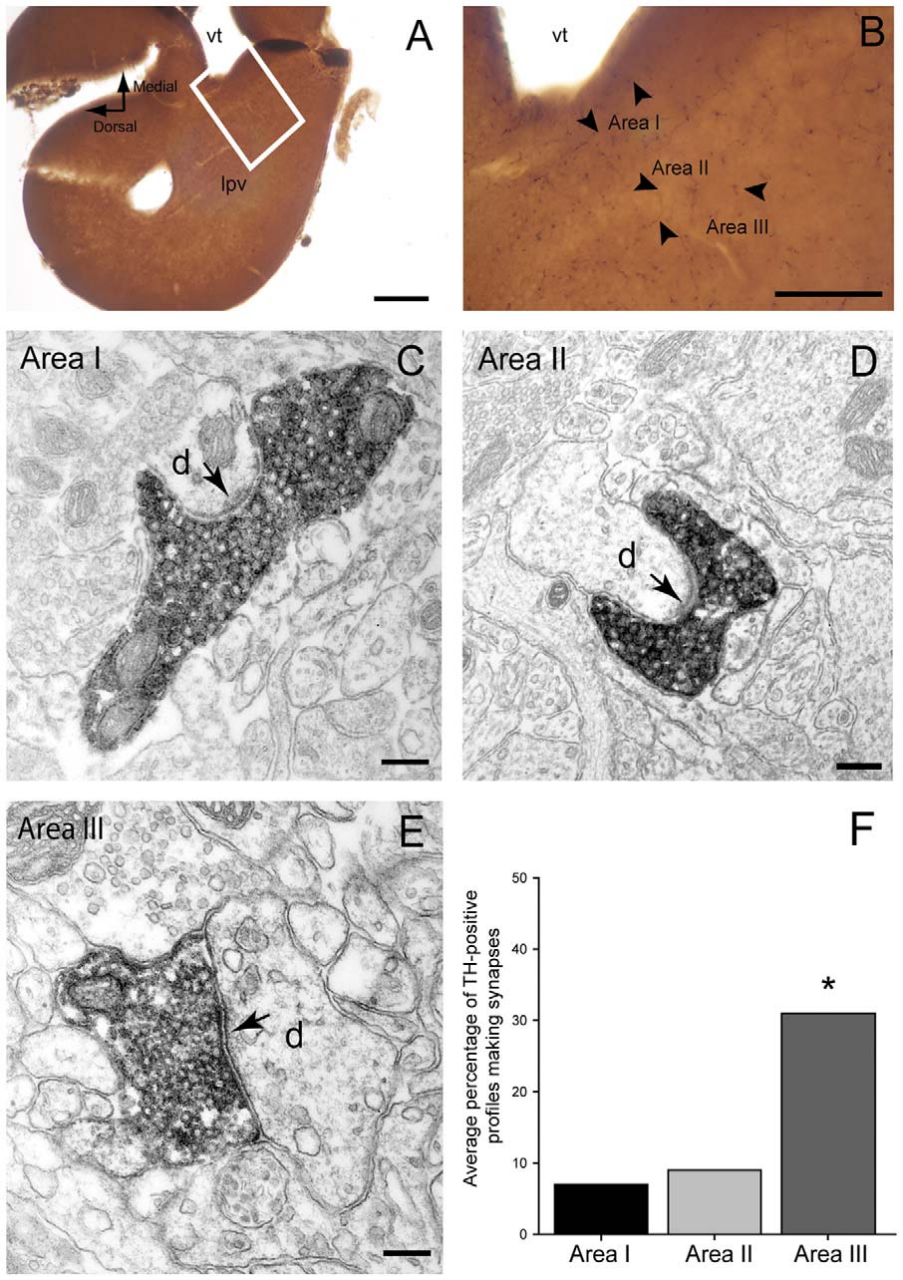
Tyrosine hydroxylase (TH) immunoreactivity in the lamprey striatum. **A and B.** Light microscope photomicrographs showing the areas (I, II and III) of the striatum that were included in the electron microscope analysis. Note the slightly higher density of TH-immunoreactive fibers (some indicated by small arrows) in Areas I and III. **C–E.** Electron micrographs of TH-immunolabeled axonal profiles forming asymmetrical synapses (arrows) with dendritic shafts (d) in Area I (C), II (D) and III (E) of the lamprey striatum. Note the densely packed vesicles and the prominent postsynaptic densities. **F.** Quantitative analysis of synaptic incidence in the three areas of striatum. The histogram shows the average percentage of TH-immunolabeled profiles which form synaptic junctions (out of a total of 75 profiles per area, n = 3). Synaptic incidence in Area III was significantly higher when compared to Area I (*x* ^2^ test, p = 0.0003) and Area II (*x* ^2^ test, p = 0.0022). Abbreviations: *vt, ventriculus medius telencephali; lpv, ventral part of the lateral pallium*. Scale bars: A: 200 µm, B: 100 µm, C–E: 200 nm.

The striatum is densely innervated by long, TH-positive axons, which are mainly distributed in areas III and I, with area II containing slightly fewer immunoreactive profiles (Figure 6B). In all areas, TH-positive profiles contained densely packed round vesicles (Figure 6C–E) distributed throughout the axon, and were commonly associated with two or more mitochondria. All immunolabeled structures that formed synaptic contacts, formed asymmetric synapses that were characterized by the presence of a long synaptic specialization, presynaptic vesicle accumulation, a thick postsynaptic density, a widened synaptic cleft and cleft material (Figure 6C–E).

In all three areas (I, II and III) the postsynaptic structures were dendrites that varied in size and shape, some of them having a stubby (Figure 6D) or spine-like appearance. We did not observe any immunopositive boutons establishing synapses of the symmetrical type (Gray's type 2) or making contact with a cell body.

The synaptic incidence analysis on the serial sections of the TH-labeled axonal profiles demonstrates that most do not form a synaptic junction within six consecutive sections. The proportion of TH-labeled axons that make synapses in area III was 31%, which was significantly higher than the synaptic incidence observed in area I (*x* ^2^ test, p = 0.0003) and area II (*x* ^2^ test, p = 0.0022), where only 7 and 9% of labeled profiles formed synapses, respectively (Figure 6F).

### The effect of D2 receptor activation on striatal neurons

In mammals and birds, selective activation of D2 receptors reduces the overall excitability of striatal projection and interneurons through modulation of intrinsic and synaptic properties [12], [31], [32]. To investigate the effects of D2 receptor activation on lamprey striatal neurons, we applied the selective D2 agonist, TNPA, during whole cell current-clamp recordings.

**Figure 7:**
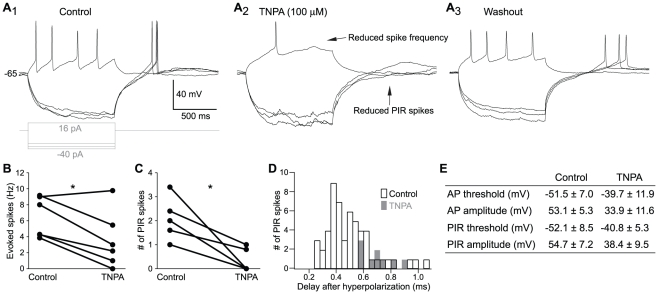
Modulation of striatal neurons by a dopamine D2 agonist. **A.** Voltage responses of a striatal neuron to 1 s long hyperpolarizing and depolarizing current injections during control (A_1_), D2 agonist application (TNPA, A_2_) and washout (A_3_). **B.** The number of evoked action potentials is reduced upon application of TNPA, measured here at twice the threshold stimulation. **C.** The average number of PIR spikes is reduced by TNPA, measured after termination of five consecutive hyperpolarizations in between −100 and −80 mV. **D.** The total number of PIR spikes and the time until the spike are reduced by TNPA, measured as in C. **E.** Table of firing properties before and during TNPA. All values presented as mean ± SD and differences were significant for all measurements apart from the AP half-width (p<0.05, student's t-test). Abbreviations: *AP, action potential; PIR, post-inhibitory rebound; TNPA, trihydroxy-N-propyl-noraporphine 123 hydrobromide hydrate*.

Depolarizing current steps from a slightly depolarized baseline of -65 mV applied to striatal neuron elicited a series of action potentials (Figure 7A_1_–A_3_). Application of 100 µM TNPA markedly reduced the number of evoked spikes (Figure 7A_2_). Comparison of the spike frequency of evoked action potential at twice the threshold current for evoking a spike revealed a marked and significant reduction in 5 of 6 neurons in the presence of TNPA (control 6.4±2.5 Hz; TNPA 3.6±3.6; p<0.05; n = 6; Figure 7B). The reduction was not affected by changing the baseline from which action potentials were evoked.

At the termination of hyperpolarizing current steps a post-inhibitory rebound (PIR) depolarization occurred with superimposed spikes (Figure 7A_1_, control). This occurs at the baseline level of −65 mV, but not at holding-potentials below −80 mV (n = 6). During TNPA application, the number of PIR spikes (Figure 7A_2_ and C) was markedly reduced (control 1.9±0.9; TNPA 0.3±0.5; p<0.05; n = 6). Moreover, when rebound spikes were seen in the presence of TNPA, they tended to occur much later after the end of the hyperpolarizing pulse (Figure 7D). TNPA also affected the size and shape of evoked action potentials and PIR spikes (Figure 7E).

## Discussion

This study represents the first description of the lamprey dopamine D2 receptor cDNA and its expression pattern in the forebrain. We revealed a strong and distinct expression of the D2 receptor mRNA in the striatum. The same region contains TH-immunoreactive synaptic terminals. Moreover, D2 receptor agonists were found to exert similar cellular effects on striatal neurons as established in mammals [12].

### The lamprey dopamine D2 receptor gene

Our analysis of the nucleotide and deduced amino acid sequence of the lamprey D2 receptor demonstrates that it is a member of the superfamily of G-protein coupled receptors and belongs to the D2 receptor subtype. The gene has two highly conserved parts that comprise all the TM domains containing the amino acids necessary for binding of dopamine [25], [26]. The unconserved intermediate part that forms the third intracellular loop differs, however, largely from other vertebrates. The longer coding sequence of the lamprey D2 receptor compared to the human long isoform, 1533 bp versus 1332 bp, is mainly due to its longer third cytoplasmic loop. A phylogenetic analysis of the cloned D2 receptor gene rooted the lamprey protein sequence at the base of the D2 receptor tree and furthest from the human D2 receptor in line with lamprey having emerged earlier than any of the other vertebrate species (approximately 560 million years ago; [8]). In mammals, birds and most teleosts, only one D2 receptor gene has been found that encodes for the two protein isoforms, the D2 long and D2 short isoforms [33], [34]. Likewise, we only identified one gene encoding for a dopamine D2 receptor, as is the case for the D1-like receptor in lamprey and hagfish [16]. However, in three teleosts (zebrafish, trout and eel), multiple D2 receptors have been identified [23], [35], [36]. Compared to other gnathostomes, an additional duplication event (3R) is thought to have occurred in the teleost lineage [37]. It is likely that in most cases this additional duplicate has been lost, whereas a subfunctionalization of ancestral functions has been proposed to explain the conservation of both D2 receptor duplicates. In lampreys, at least one duplication event is thought to have occurred, but according to our results the receptor analyzed clearly corresponds to the mammalian D2 subtype. The D2 receptor is likely to be rather conserved in the vertebrate lineage even in teleosts, where the ancestral functions were maintained despite the existence of additional D2 receptors, although additional analyses are still necessary to confirm this assumption.

### Expression of D2 receptors in the forebrain

In mammals, a clear difference between the D1 and D2 receptor expressing striatal neurons is evident at the molecular, anatomical, and physiological levels (for review see [12]). The high levels of D2 mRNA receptor expression in a subpopulation of striatal cells, as well as the absence of D2 expression in striatal cells of the direct pathway, indicate that also in lamprey, there is a segregation between these two cell-types. This is further supported by our recent studies that pallidal GPi neurons are mainly apposed by substance P-positive fibers/terminals, whereas GPe neurons are apposed by enkephalin-positive structures and that SNr projecting striatal cells are substance P-immunoreactive [6], [7].

High levels of D2 receptor expression were also observed in the preoptic area, habenula, mammillary region, hypothalamus, and thalamus. In all other vertebrates investigated, these regions are rich in D2 receptor expression (e.g. [22], [23], [35], [38], [39]) which gives further evidence that our cloned D2 receptor is indeed, a member the D2 receptor subfamily. A small region of the preoptic area, the magnocellular preoptic nucleus, lacked D2 expression, as is the case in the trout [35].

Although the dopamine D2 and D3 receptors belong to the same class of dopamine receptors their expression pattern in the vertebrate brain is very different. Overall, the D2 receptor is by far the more highly expressed subtype and is found in all major brain areas receiving dopaminergic projections, particularly the dorsal striatum. The D3 receptor is not only expressed at a lower levels but it also shows a fairly restricted pattern of expression (e.g. islands of Calleja, nucleus accumbens and cerebellum) [29], [40], [41]. The robust general expression pattern of the D2 receptor in lamprey brain further supports our conclusion that it belongs to the D2-subfamily.

### Dopaminergic terminals in striatum

The striatum in lampreys receives a dense dopaminergic innervation that originates in the nucleus tuberculi posterioris in the diencephalon [9]. The general distribution pattern of dopamine-immunoreactive neurons in this nucleus is similar to that obtained using antibodies against aromatic L-amino acid decarboxylase or TH [9], [30], [42] and furthermore this nucleus does not express the enzymes involved in the synthesis of noradrenaline or adrenaline [42] implying that the neurons are dopaminergic.

The present immunocytochemical findings confirm and extend previous findings relating to TH innervation of the lamprey striatum and demonstrate that TH-immunoreactive axonal profiles are densely packed with synaptic vesicles and form typical asymmetric synaptic specializations. Thus, similar to mammals, the lamprey striatum receives a dense innervation from TH-positive fibers. However, there are differences in the ultrastructural features in terms of their size, type of specialization and distribution of postsynaptic targets. In rodents, TH-positive boutons form symmetrical synapses (Gray's type 2) with both spines and dendritic shafts and occasionally with cell bodies [43]–[45]. Their synapses are very small and membrane specializations are often difficult to discern. In contrast, in the lamprey striatum dopaminergic terminals form exclusively asymmetric synapses with dendrites which, in addition, have large and prominent synaptic specializations. Furthermore, our quantitative analysis shows that around 30% of labeled TH-positive profiles observed in Area III form synaptic contacts (when examined in 6 serial sections), whereas profiles located in Areas I and II show a significantly lower synaptic incidence (Figure 6F). Although this degree of synaptic incidence in Areas I and II is similar to that observed in mammals, the synaptic incidence in Area III is higher and may suggest that dopaminergic transmission has different characteristics in this region. It is interesting to note that a single-cell analysis of intracellularly labeled Area II striatal neurons [10] revealed that their dendrites branch more profusely in Area III, i.e. the region in which dopaminergic axons are most likely to form synapses. Overall, our study reveals new aspects of the distibution and fine structural features of the dopaminergic system of the lamprey brain. Furthermore, our data provide the structural basis to support the modulatory role of dopamine within this area and thereby its contribution to basal ganglia functions in the lamprey brain.

### Modulation of dopamine in the striatum

D2 receptor activation modulates neuronal excitability through inhibition of voltage sensitive Ca^2+^ channels, inactivation of Na^+^ channels and activation of K^+^ channels [29], [46], [47]. In the present study we observed a marked reduction of discharge in lamprey striatal neurons upon D2 receptor activation, a finding that is consistent with results from reptiles, birds and mammals [31], [32], [48]. The D2 receptor agonist, TNPA, shifted the threshold to more depolarized levels for both action potentials and PIR spikes, as well as reducing their amplitudes. This is similar to observations in mammals where D2 activation appears to enhance slow inactivation of Na^+^ channels, an effect that also contributes to the reduction in the PIR response in cholinergic interneurons [49]. In mammalian striatal projection neurons, a reduced excitability has also been associated with a negative modulation of the L-type Ca_v_ 1.3 Ca^2+^ channel [48], [50]. In the lamprey spinal cord, a depression of Ca_v_ 1.3 Ca^2+^ channels by D2 receptor activation has also been described [51]. This is consistent with our findings, where application of TNPA profoundly reduces the number of PIR spikes. The fact that PIR spikes were almost only elicited upon hyperpolarization from a depolarized baseline around −65 mV or more, further suggests Ca_v_ 1.3 channels to be involved since this is within their activation range [52].

In mammals, D2 receptors are expressed in neurons that project to the GPe, as part of the indirect pathway [53]. Selective acivation of the indirect pathway medium spiny neurons leads to an inhibition of movements [54], presumably by increasing the inhibitory output of the GPi/SNr [12]. The indirect pathway has been oberserved in lamprey and may represent a common vertebrate mechanism for inhibiting actions [7], [54]. Application of dopamine or D2 receptor antagonists cause a decrease in the excitability of mammalian indirect pathway medium spiny neurons [48], [55], whereas dopamine increases the excitability of medium spiny neurons that express the D1 receptor and project directly to the GPi/SNr [56], [57]. The dicotomous effects of dopamine on these pathways is therefore believed to bias the network to promote actions, by decreasing the excitability of the indirect pathway that suppresses actions and by increasing the excitability of the direct pathway that promotes actions. Our results show that lamprey striatal neurons express the D2 receptor and that D2 receptor agonists decrease the excitability of striatal neurons. This suggests that the direct and indirect pathways are not only conserved throughout vertebrate phylogeny as a common mechanism for action selection but that dopmine will play a similar role in modulating at least D2 receptor expressing neurons to bias the network for action selection.

### Conclusion

The present identification of a functional dopamine D2 receptor and the structural basis for the modulatory role of dopamine in the lamprey striatum gives additional evidence for the presence of an indirect pathway and that the ancestral basal ganglia circuitry is evolutionary conserved. The resemblances in gene structure between different vertebrate D2 receptors furthermore show that the fundamental function of these receptors is a conserved feature.

## Material and Methods

### Ethical statement

Experiments were performed on a total of 32 adult river lampreys (*Lampetra fluviatilis*) of either sex. The experimental procedures were approved by the local ethical committee (Stockholm's Norra Djurförsöksetiska Nämnd) and were in accordance with *The Guide for the Care and Use of Laboratory Animals* (National Institutes of Health, 1996 revision). During the investigation, every effort was made to minimize animal suffering and to reduce the number of animals used.

### Molecular cloning and sequencing of the lamprey dopamine D2 receptor

#### RNA isolation

Animals (n = 9) were deeply anesthetized in tricane methane sulfonate (MS-222; 100 mg/L; Sigma-Aldrich, St. Louis, MO, USA) diluted in fresh water and the brains were quickly removed and frozen on dry ice. Total RNA was extracted using Trizol reagent (Invitrogen, La Jolla, CA, USA) or RNeasy Mini Kit (Qiagen, Valencia, CA, USA) and quantified by spectrophotometry using NanoDrop® ND-1000 spectrophotometer (NanoDrop Technologies, Wilmington, DE, USA). The integrety and purity of the RNA preparations were analysed by means of gel electrophoresis on Experion bioanalyzer (Bio-Rad Laboratories, Hercules, CA, USA). Only RNA with RQI values between 9.9 and 10 (max value 10) was used for cDNA synthesis.

#### cDNA synthesis

The first-strand cDNA synthesis from total RNA was performed with RETROscript reverse transcriptase (Ambion Inc, Huntingdon, UK) and Oligo (dT) or Random decamers. Identical results were obtained with both.

#### PCR amplification

For polymerase chain reaction (PCR) amplification, degenerate oligonucleotide primers were designed based on alignment of the human dopamine D2 receptor (GenBank accession number NM_000795) with the lamprey (*Petromyzon marinus* WGS) genome sequences deposited in the trace archives at the NCBI (National Center for Biotechnology Information, Bethesda, MD, USA). Primers (see Table 1) were designed to amplify different parts of the predicted lamprey D2 receptor sequence using Primer3 software (Whitehead Institute/Massachusetts Institute of Technology, Boston, MA) and purchased from Invitrogen. In addition, we used the SMART RACE cDNA amplification kit (Clontech Laboratories, Palo Alto, CA; see below) to obtain the unconserved amino terminus and to verify the start and stop codons. All primers had a GC content between 50 and 61%. The amplified cDNA fragments were cleaned directly with the MinElute PCR Purification kit (Qiagen) or cloned into a pCR®II-TOPO® vector (Invitrogen, see below) and then confirmed by nucleotide sequencing (KIGene, Core Facility at Karolinska Institutet, Stockholm, Sweden).

#### 5′- RACE

First strand cDNA was synthesized from total RNA using a modified lock-docking oligo (dT) primer (5′-RACE cDNA synthesis primer) and SMARTer IIA oligo, following the manufacturer's instructions (Clontech). The first strand cDNA was used as a template for PCR amplification with a gene specific primer (Table 1) and a universal primer mix containing the complementary sequence to the SMART oligo. The 5′-RACE PCR was performed in 50 µL reactions using Clontech Advantage 2 Polymerase Mix following the manufacturer's instruction (Clontech). The amplified fragment was cloned using the TOPO TA Cloning® Kit with pCR®II-TOPO® vector and One Shot® TOP10 Chemically Competent cells following the manufacturer's instructions (Invitrogen). Transformed bacteria were grown overnight at 37°C on LB agar plates. Twelve bacteria colonies were selected and grown overnight in Terrific Broth (Sigma) at 37°C. Plasmid DNA purification was subsequently performed using the Bio-Rad Quantum Prep Plasmid Miniprep Kit (Bio-Rad Laboratories, Sundbyberg, Sweden). Purified DNA fragments were confirmed by nucleotide sequencing (KIGene).

#### 3′-RACE

First strand cDNA was synthesized from total RNA using a modified lock-docking oligo (dT) primer (3′-RACE cDNA synthesis primer) according to the manufacturer's instructions (Clontech). For the gene specific primers used see Table 1.

### Sequence analysis

Sequence analysis and comparisons were made with the Basic Local Alignment Search Tool (BLAST) on NCBI and/or the CLC Sequence Viewer 6.4. The start site of translation for the putative lamprey dopamine D2 receptor was determined based upon the following: *i*) the putative start codon being embedded in a consensus motif of a ribosomal binding site (see [24], *ii*) the open reading frame using ORF finder at NCBI (http://www.ncbi.nlm.nih.gov) and *iii*) by BLAST analysis (comparing the putative lamprey protein sequence with D2 receptor proteins of other known vertebrate species).

Transmembrane domains predictions were made with Octopus (http://octopus.cbr.su.se/). Putative N-glycosylation and phosphorylation sites were analyzed using PredictProtein-Sequence Analysis, Structure and Function Prediction (http://www.predictprotein.org).

The amino acid sequence of the lamprey dopamine D2-like receptor was aligned with homologous D2 receptor sequences from other known vertebrate representatives using BLAST and Geneious (http://www.geneious.com). Dopamine D3 and D4 receptor sequences were also included in the analysis. A phylogenetic tree was constructed with the neighbor-joining method from this alignment. Detailed analysis and alignments were also made with *Homo sapiens* (NP_000786.1), *Mus musculus* (NP_034207.2), *Danio rerio* (NP_898891.1) *and Anguilla anguilla* (ABH06893.1) D2 receptor proteins using CLC Sequence Viewer 6.

### Probes for *in situ* hybridization

Templates for *in vitro* transcription were prepared by PCR amplification. A 660 base pair (bp) fragment (see Figure 2A) was obtained using 5′-TGCTCATATGCCTCATCGTC-3′ forward and 5′-TCAAGCTTTGCACAATCGTC-3′ reverse primers. The amplified cDNA fragment was cloned into a pCR®II-TOPO® vector (Invitrogen), cleaned and confirmed by nucleotide sequencing (KIGene). Linearized plasmids (1 µg) were used to synthesize [^35^S] UTP- and digoxygenin (DIG)-labeled riboprobes. *In vitro* transcription was carried out using the MAXIscript™ SP6/T7 kit (Applied Biosystems, Uppsala, Sweden) and [α35-S]UTP (NEG039H; Perkin Elmer, Upplands Väsby, Sweden) or DIG RNA Labeling Mix (Roche Diagnostics, Nutley, NJ, USA) according to the manufacturer's instructions. The transcripts were purified using NucAway™ spin columns (Applied Biosystems). Sense probes were used as negative controls.

### *In situ* hybridization

Adult river lampreys (n = 12) were deeply anesthetized in MS-222 (100 mg/L) diluted in fresh water. Brains were quickly removed and fixed in 4% paraformaldehyde or 4% formaldehyde in 0.01 M phosphate buffered saline (PBS) overnight at 4°C. The tissue was cryoprotected in 20% sucrose in 0.01 M PBS overnight and 20 µm thick serial, transverse cryostat sections were cut through the brain and stored at −80°C until further processing. In each *in situ* hybridization that we performed one series of sections was used for the anti-sense probe and the adjacent series was used for the sense probe.

#### *In situ* hybridization with ^35^S-UTP-labeled riboprobes

The sections were left at room temperature for 30 min, washed in 0.01 M PBS, treated with 0.05 M HCl for 10 min, washed in DEPC-treated water and then 0.01 M PBS, acetylated in 0.25% acetic anhydride in 0.1 M triethanolamine (pH 8.0) for 15 min, washed in 0.01 M PBS and dehydrated in 70%, 80% and 100% ethanol (2 min each). Sections were air dried and prehybridized (50% deionized formamide (pH 5), 50 mM Tris-HCl (pH 7.6), 25 mM EDTA (pH 8.0), 20 mM NaCl, 0.25 mg/ml yeast tRNA, 2.5× Denhardt's solution) for 4 h at 55°C. Sections were then hybridized overnight (14–16 h) at 55°C with labeled probes diluted to a final concentration of 1.0× 10^6^ cpm/200 µL in a solution containing 50% deionized formamide, 0.3 M NaCl, 20 mM Tris-HCl (pH 7.6), 5 mM EDTA (pH 8.0), 10 mM PBS, 0.2 mM dithiothreitol, 0.5 mg/ml yeast tRNA, 0.1 mg/ml poly-A-RNA, 10% dextran sulfate, and 1× Denhardt's solution. After hybridization, the slides were rinsed in 1× standard saline citrate (SSC), 0.01% sodium dodecyl sulfate (SDS: 15 min); 1× SSC, 0.01% SDS (30 min); 50% formamide/0.5× SSC (1 h); 1× SSC, and 0.01% SDS (15 min) at 55°C with continuous shaking. The sections were then treated with 1 µg/mL RNase A (Roche, Diagnostics) in RNase buffer (0.5 M NaCl, 10 mM Tris-HCl, 5 mM EDTA, pH 8.0) for 1 h at 37°C. After two additional washes in 1× SSC, 0.01% SDS for 30 min, the sections were dehydrated in ascending alcohol series and air-dried. Sections were placed against β-Max film (VWR, Sweden) and stored at room temperature for 3 to 5 days. Films were developed in Kodak D19 developer for 2 min and in Kodak Unifix liquid fixative for 5 min. Films were scanned with Epson Perfection 4990 Photo scanner as gray scale film. The gray scale was converted to pseudocolors using ImageJ (NIH ImageJ version 1.45, U.S. National Institutes of Health).

#### *In situ* hybridization with DIG-labeled riboprobes

Sections were handled as above excluding the HCl treatment. After prehybridization (50% formamide, 5×SSC pH 7.0, 5×Denhardts's, 500 µg/mL salmon sperm DNA, 250 µg/mL yeast RNA) for 2–4 h at 60°C, DIG-labeled sense and anti-sense riboprobes were prepared and added to the hybridization solution to a final concentration of 500 ng/mL and hybridized overnight at 60°C. An RNAse treatment (20 µg/mL in 2×SSC) was performed for 30 min at 37°C following stringent washes in SSC (Applied Biosystems). After additional washes in maleic acid buffer (pH 7.5) the sections were incubated overnight at 4°C in alkaline phosphatase labeled sheep anti-DIG antibody (1∶2000; Roche Diagnostics) in 10% heat inactivated normal goat serum (NGS; Vector Laboratories, Burlingame, CA). The alkaline phosphatase was visualized using BCIP/NBT (Roche Diagnostics) in TRIS buffer pH 9.5 without the addition of MgCl_2_. The sections were dehydrated and mounted in DPX (Sigma). Photomicrographs were taken in an Olympus BX51 microscope using the image acqusition program Cell^A^ and adjusted for brightness and contrast with Adobe Photoshop CS4.

### Retrograde tracing

Animals (n = 2) were deeply anesthetized in MS-222 in ice-cooled oxygenated HEPES buffered physiological solution (in mM: 138 NaCl, 2.1 KCl, 1.8 CaCl_2_, 1.2 MgCl_2_, 4 glucose, and 2 HEPES; pH 7.4). 50–200 nL 20% neurobiotin (Vector Laboratories) was pressure injected unilaterally into the lamprey homologue of the SNr, located in the dorsolateral isthmic region. All injections were made with glass micropipettes (borosilicate, OD = 1.5 mm, ID = 1.17 mm). Following the injections, the animal were returned to their aquarium for 24 h. Following anesthesia, fixation, sectioning of the brain and *in situ* hybridization of striatal sections with DIG-labeled D2 receptor riboprobes (see above), the tissue was incubated with Cy3-streptavidin (1∶1000; Jackson Immunoresearch, West Grove, PA) to reveal the neurobiotin, rinsed in PBS and mounted in glycerol containing 2.5% DABCO (Sigma-Aldrich). The total number of retrogradely labeled cells in the striatum was counted in two animals. Photomicrographs were taken in an Olympus BX51 microscope with an Olympus XM10 CCD camera (black and white), using the image acqiusition program Cell^A^ and adjusted for brightness and contrast with Adobe Photoshop CS4.

### Preparation and analysis of tissue for electron microscopy

#### Tissue preparation

Following anesthesia, lamprey brains (n = 3) were immersion-fixed overnight in 4% formaldehyde, 14% saturated picric acid solution and 0.1% glutaraldehyde in 0.1 M phosphate buffer (PB; pH 7.4) at 4°C, and then transferred to 0.1 M PB until processing. The brains were embedded in 5% agar and sectioned at 50 µm in the coronal plane using a vibrating microtome (VT1000S; Leica Microsystems, Wetzlar, Germany).

#### Immunohistochemistry

The sections were incubated in a cryoprotectant solution (0.05 M phosphate buffer, 25% sucrose, 10% glycerol) overnight, then freeze-thawed twice in liquid nitrogen in order to increase penetration of the reagents. The sections were washed thoroughly and then incubated in 10% NGS (Vector Laboratories) in PBS for 2 h at room temperature. All sections were immunolabeled to reveal TH-containing structures using a rabbit anti-TH antibody (1∶500 in NGS-PBS; Millipore AB152). The sections were incubated overnight at room temperature, washed in PBS and incubated at 4°C with a biotinylated goat anti-rabbit secondary antibody (1∶300 in NGS-PBS; Vector Laboratories) for about 16 h. This was followed by incubation in an avidin-biotin-peroxidase complex (ABC Elite; Vector Laboratories) for 3–4 h at room temperature. The sections were then washed in PBS followed by washes in Tris-buffer (0.5 M, pH 7.6; TB). The sections were pre-incubated in diaminobenzidine (DAB 0.025% in TB) with shaking for 15 min and the peroxidase reaction was initiated by the addition of H_2_O_2_ to a final concentration of 0.01%. The reaction was allowed to continue for 3–5 min and stopped by several washes in TB and then PB. The sections were postfixed in 1% osmium tetroxide in PB for 15–20 min and dehydrated as described previously [45]. Following absolute ethanol, sections were washed twice in propylene oxide (Sigma) for 15 min and placed into resin overnight at room temperature (Durcupan ACM, Fluka, Gillingham, UK). They were then mounted in resin on glass slides and the resin was polymerized at 60°C for 48 h.

#### Electron microscopic analysis

It has previously been shown [9] that striatal neurons and neuropil are organized into three layers, characterized as follows: Area I is defined by the neuropil located in the most medial part of the striatum which faces the *ventriculus medius telencephali*; Area II contains most of the striatal cell bodies arranged as a single band. These neurons possess two major processes that extend and branch in opposite directions, medially towards Area I and laterally towards Area III, which also contains scattered cell bodies. Each of these regions were investigated for the presence of TH-immunoreactive synaptic terminals and the nature of the postsynaptic targets.

Regions of the sections that contained Areas I, II and III (Figure 7 A, B) were cut out from the slide and re-embedded in Durcupan blocks. Serial sections (50–60 nm) were cut using an ultramicrotome (Leica EM UC6, Leica Microsystems) and collected on pioloform-coated, single-slot copper grids (Agar Scientific, Stansted, UK). To improve contrast, the ultrathin sections were lead-stained for 4–5 min and then examined in a Philips CM100 electron microscope.

TH-immunoreactive structures were identified by the electron dense peroxidase reaction product that adhered to the internal surface of the plasmalemma and the outer membrane of organelles and axonal profiles were identified by the presence of synaptic vesicles and often mitochondria. TH-immunolabeled profiles located in the three different areas were noted and digitally recorded at different magnifications using a Gatan CCD camera. Each profile was examined in each of the six serial sections to determine whether they form of a synaptic specialization and to characterize the nature of the postsynaptic target. Any other immunopositive axonal profile seen in a subsequent serial section was also noted and analyzed in all sections. This process was repeated on sections from different grids until 25 TH-immunopositive profiles were analyzed per area per animal. In total, 225 TH-positive profiles were included in the analysis. A total of aproximately 40,000 square micrometer were examined per animal.

The digital images were analyzed using Image J, and they were adjusted for contrast and brightness using Adobe Photoshop CS3. Synapse formation throughout the serial sections was scored for each TH-positive axonal profile. For each such synapse, the postsynaptic target was characterized. All synapses observed were of the asymmetrical type (Gray's type 1) and were characterized as such by the presence of presynaptic vesicle accumulation, a thick postsynaptic density, a widened synaptic cleft and cleft material. Structures which did not fulfill these criteria were not considered as synaptic junctions.

Statistical analyses were performed using the statistical software R (version 2.13.1). Data were presented as means and standard deviations and comparisons made using chi-squared test with Yates continuity correction. Tests with p<0.05 were considered significant.

### Electrophysiology in lamprey striatal slices

Acute striatal brain slices were prepared from anesthetized (MS-222, 1 mg/100 mL) lamprey as described in Ericsson et al. [58]. Brains were quickly dissected out from decapitated lampreys in ice-cold artificial cerebrospinal fluid (aCSF) of the following composition (in mM): NaCl 125, KCl 2.5, NaH_2_PO_4_ 1.25, MgCl_2_ 1, glucose 10, CaCl_2_ 2 and NaHCO_3_ 25. Sectioning of the brain was performed with a tissue chopper (Vibratome 800 tissue Chopper, Leica Microsystems AB, Sweden) to obtain coronal slices of 350 µm thickness. The slices were transferred to a recording chamber and continuously perfused with aCSF (pH 7.4) that was oxygenated with 95% O_2_ and 5% CO_2_. The chamber was kept at 5–8°C with a Peltier cooling block (Elfa, Stockholm, Sweden). Slices were left to recover for at least one hour before patch clamp experiments were commenced. Recordings were performed in whole-cell current- or voltage-clamp mode with patch pipettes of 5–12 MΩ filled with (in mM): 105 K-gluconate, 30 KCl, 10 HEPES, 4 Mg-ATP, 0.3 Na-GTP and 10 phosphocreatine sodium salt (osmolarity 275 mOsm). Pipettes were prepared from borosilicate glass microcapillaries (Harvard Apparatus, Kent, UK) using a three-stage puller (Model P-97, Sutter Instruments, Novato, CA, USA). Neurons were visualized with DIC/infrared optics (Olympus BX51WI, Tokyo, Japan) and pipettes advanced with remote-controlled micromanipulators (Luigs & Neumann, Ratingen, Germany). An Axoclamp 2B amplifier (Molecular Devices Corp., CA, USA) was used together with a HS-2A headstage (Molecular Devices Corp.) for recordings and signals digitized (ITC-18, HEKA, Lambrecht, Germany) at 10–50 kHz before storage on a PC. Bridge balance and pipette capacitance compensation were adjusted on the amplifier and membrane potential values were corrected for the liquid junction potential. Data was acquired and analyzed using Igor software (version 6.03, WaveMetrics, Portland, USA).

The dopamine D2 receptor agonist TNPA (R(−)-2,10,11-Trihydroxy-N-propyl-noraporphine 123 hydrobromide hydrate; Sigma-Aldrich), was freshly prepared as 100 mM in 99.5% ethanol before dilution to 100 µM in aCSF. TNPA was then bath-applied through the perfusion system.

Statistical analyses were performed with GraphPad Prism Software (GraphPad Software, San Diego, CA, USA) and comparisons between means were made with two-tailed paired t-tests. Results are presented as mean ± standard deviation (SD).

## Supporting Information

Figure S1 **Nucleotide and deduced amino acid sequences of the lamprey dopamine D2 receptor.** The coding region is 1533 base pair long and its deduced amino acid sequence spans 511 amino acids. The numbering of the deduced amino acid sequence begins with the first methionine of the open reading frame, and is shown to the right of each line. The nucleotide numbers are shown to the left of each line. The untranslated regions are shown in smaller fonts. Putative N-glycosylation sites, blue squares; protein kinase C phosphorylation sites, green squares; cAMP phosphorylation site, pink square; Casein kinase II (CKII) phosphorylation site, orange squares.
(TIF)
