# Supplementary figures and images for: The Dopamine D2 Receptor Gene in Lamprey, Its Expression in the Striatum and Cellular Effects of D2 Receptor Activation
Source: PLoS One. 2012 Apr 26;7(4):e35642. doi: 10.1371/journal.pone.0035642 (PMC3338520; doi:10.1371/journal.pone.0035642)

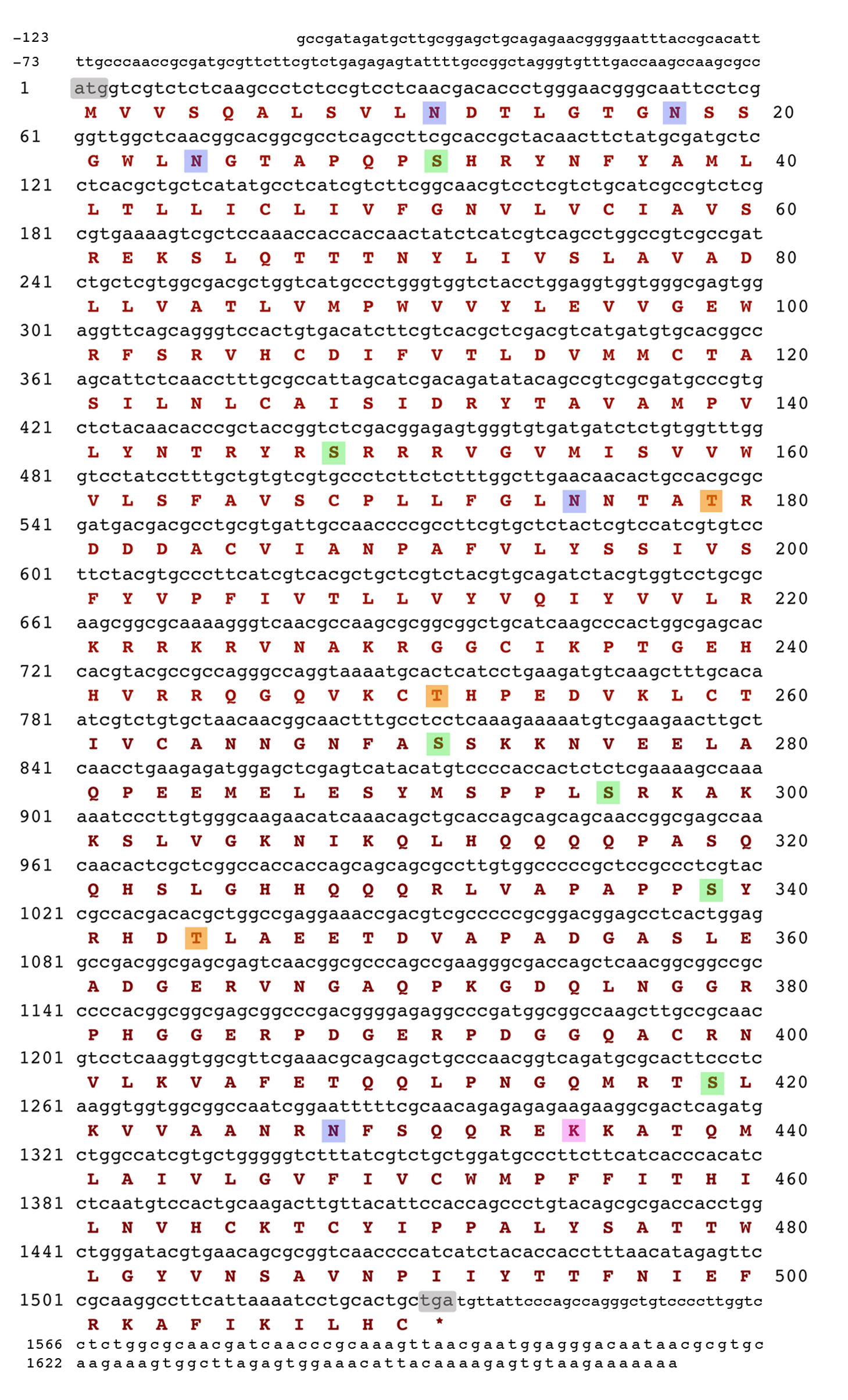

Supplement: Figure S1 — Nucleotide and deduced amino acid sequences of the lamprey dopamine D2 receptor. The coding region is 1533 base pair long and its deduced amino acid sequence spans 511 amino acids. The numbering of the deduced amino acid sequence begins with the first methionine of the open reading frame, and is shown to the right of each line. The nucleotide numbers are shown to the left of each line. The untranslated regions are shown in smaller fonts. Putative N-glycosylation sites, blue squares; protein kinase C phosphorylation sites, green squares; cAMP phosphorylation site, pink square; Casein kinase II (CKII) phosphorylation site, orange squares. (TIF) [file pone.0035642.s001.tif]
